# Mapping the evidence on interventions that mitigate the health, educational, social and economic impacts of child marriage and address the needs of child brides: a systematic scoping review

**DOI:** 10.1080/26410397.2024.2449310

**Published:** 2025-01-08

**Authors:** Shatha Elnakib, Ahmed K. Ali, Kate Mieth, Venkatraman Chandra-Mouli

**Affiliations:** a Assistant Scientist, International Health Department, Johns Hopkins Bloomberg School of Public Health, Baltimore, MD, USA.; b Adolescent and Youth Sexual and Reproductive Health and Rights Consultant, Department of Sexual and Reproductive Health and Research (which includes the Human Reproduction Programme), World Health Organization, Geneva, Switzerland; c Associate, Johns Hopkins Bloomberg School of Public Health, Baltimore, MD, USA; d Formerly Scientist, Department of Sexual and Reproductive Health and Research (which includes the Human Reproduction Programme), World Health Organization, Geneva, Switzerland

**Keywords:** child marriage, scoping review, adolescent, interventions, low- and middle-income countries

## Abstract

More than 650 million women alive today were married as children. Relative to efforts to prevent child marriage, efforts to support child brides have received much less attention. This review set out to map and describe interventions that support child brides. We performed a scoping review using seven electronic databases coupled with a grey literature search in January 2022. Data were extracted using a piloted extraction tool and findings were reported in narrative synthesis. A total of 34 projects were included in our review. Most projects focused on improving sexual and reproductive health (SRH) knowledge and behaviours among child brides, which was often achieved through a combination of SRH education, counselling and information provision, along with linkages to SRH services. Some interventions were health facility-based and aimed at improving responsiveness of health service providers to the needs of child brides. Very few described economic interventions as one component of a broader health intervention, and only three interventions focused on improving girls’ educational outcomes. We also note the paucity of media-based interventions, despite their popularity among adolescents. Over time, interventions addressing the needs of child brides have increased, but the preponderance of evidence has focused on SRH interventions, with interventions that couple education with adolescent-friendly health services demonstrating promise. Interventions addressing other areas of health and social wellbeing of this group, such as mental health, sexual health, and economic independence, have been overlooked in comparison. The review highlights the need for additional empirical evidence on what works to support child brides.

## Plain Language Summary

Women and girls who are married under age 18 have specific vulnerabilities and unique needs for interventions and services. While child marriage prevention has received substantial attention over the past decade, as evidenced by several systematic reviews investigating what works to prevent child marriage, very few reviews have been done to assess the effectiveness of interventions that address the needs of child brides. This review takes stock of programmes and interventions that have a specific focus on meeting the health and social needs of child brides. Our review finds that most published interventions focus on improving sexual and reproductive health outcomes in this population, and few interventions target the mental health needs and social wellbeing of this group, despite evidence that child brides have worse mental health outcomes and are more likely to experience adverse social outcomes like intimate partner violence. Programming to enhance women and girls’ economic wellbeing and education are also underrepresented in this body of literature. With a critical mass of studies investigating sexual and reproductive health outcomes in this group, we can conclude that programmes which empower child brides with knowledge and SRH information when coupled with referral and accompaniment to adolescent and women friendly health services show serious potential for improving outcomes.

## Résumé

Plus de 650 millions de femmes vivant aujourd’hui ont été mariées alors qu’elles étaient encore enfants. Par rapport aux activités destinées à prévenir les mariages d’enfants, les efforts visant à soutenir les fillettes mariées ont reçu beaucoup moins d’attention. Cet examen a entrepris de recenser et de décrire les interventions qui épaulent les épouses enfants. En janvier 2022, nous avons effectué une étude de portée à l’aide de sept bases de données électroniques associée à une recherche dans la littérature grise. Les données ont été extraites en utilisant un outil d’extraction piloté et les résultats ont été présentés dans une synthèse narrative. Au total, 34 projets ont été inclus dans notre examen. La plupart des projets portaient essentiellement sur l’amélioration des connaissances et des comportements en matière de santé sexuelle et reproductive (SSR) chez les jeunes épouses, ce qui a souvent été réalisé grâce à une combinaison d’éducation, de conseil et d’information en matière de SSR, parallèlement à des liens avec les services de SSR. Certaines interventions avaient été menées dans des centres de santé et visaient à améliorer la réactivité des prestataires de services de santé aux besoins des filles mariées. Très peu d’entre elles comprenaient les interventions économiques comme l’une des composantes d’une intervention sanitaire plus large, et trois interventions seulement se centraient sur l’amélioration des résultats scolaires des filles. Nous avons également noté la rareté des interventions basées sur les médias, en dépit de leur popularité auprès des adolescents. Au fil du temps, les interventions visant à répondre aux besoins des filles mariées se sont multipliées, mais la plupart des données probantes se sont concentrées sur les interventions de SSR, les activités qui associaient l’éducation à des services de santé adaptées aux adolescentes se révélant prometteuses. En comparaison, les interventions visant d’autres domaines de la santé et du bien-être social de ce groupe, comme la santé mentale, la santé sexuelle et l’indépendance économique, ont été négligées. L’étude met en lumière la nécessité de disposer de données empiriques supplémentaires sur les mesures efficaces pour soutenir les filles mariées.

## Resumen

Más de 650 millones de mujeres vivas actualmente se casaron cuando eran niñas. En comparación con los esfuerzos por evitar el matrimonio infantil, los esfuerzos por apoyar a las novias niñas han recibido mucho menos atención. Esta revisión pretendía mapear y describir las intervenciones que apoyan a las novias niñas. En enero de 2022, realizamos una revisión de alcance utilizando siete bases de datos electrónicas junto con una búsqueda de la literatura gris. Extrajimos datos utilizando una herramienta de extracción piloteada y reportamos los hallazgos en síntesis narrativa. Incluimos en nuestra revisión un total de 34 proyectos, la mayoría centrada en mejorar los conocimientos y comportamientos de novias niñas relacionados con la salud sexual y reproductiva (SSR), lo cual a menudo se logró por medio de una combinación de educación, consejería y provisión de información sobre SSR, junto con vínculos a servicios de SSR. Ciertas intervenciones se realizaron en establecimientos de salud y pretendían mejorar la capacidad de respuesta de los prestadores de servicios de salud a las necesidades de las novias niñas. Muy pocos describieron las intervenciones económicas como un componente de una intervención sanitaria más amplia, y solo tres intervenciones se centraron en mejorar los resultados educativos de las niñas. Cabe señalar la escasez de intervenciones basadas en los medios, a pesar de su popularidad entre adolescentes. Las intervenciones que abordan las necesidades de las novias niñas han aumentado a lo largo del tiempo, pero la preponderancia de evidencia se ha enfocado en intervenciones de SSR, y las intervenciones que combinan la educación con servicios de salud amigables a adolescentes demuestran estar a la altura de lo que prometen. En cambio, se ha hecho caso omiso de intervenciones que abordan otras áreas de la salud y el bienestar social de este grupo, tales como salud mental, salud sexual e independencia económica. La revisión destaca la necesidad de obtener más evidencia empírica de lo que funciona para apoyar a las novias niñas.

## Introduction

Almost one in five girls globally are married before their 18th birthday, and no world region is projected to meet the Sustainable Development Goal of eliminating child marriage by 2030. ^1,2^ Driven by poverty, entrenched gender inequality and harmful gender norms, child marriage endangers girls’ rights to health and education and limits their opportunities to reach their full potential^3^ Married girls face a myriad of challenges, ranging from gender-based violence and exposure to unsafe sex, to social isolation and restricted agency.^4,5^ Furthermore, in many settings, children of very young mothers experience a greater risk of neonatal death, low birth weight, under nutrition, and late physical and cognitive development.^6,7^

Despite increasing attention directed to the topic and renewed urgency to eliminate the practice, child marriage continues to be prevalent in many regions of the world and there are currently around 650 million child brides whose health and wellbeing have been compromised by child marriage.^2^ The sheer number of married adolescent girls today underscores the need to strengthen policy and programmatic efforts both to prevent child marriage and to respond to the health and social needs of married girls.

Yet, most policy and programmatic responses to child marriage and research on the topic have focused on prevention of child marriage alone.^5,8,9^ In contrast, responses to meet the health and social needs of girls who are already married are limited, as is research on the effectiveness of such interventions.^8–10^ In 2019, a convening of experts – organised by the World Health Organization, the UNICEF-UNFPA Global Programme to End Child Marriage, and Girls not Brides – focused on identifying child marriage research priorities noted a glaring gap in research on how best to support child brides.^8^ Particularly noted was the lack of “evidence on the effectiveness, cost, and cost-effectiveness of a critical package of interventions” that can be used to address the needs of this often-overlooked group.^8^

No previous attempt has been made to map the research landscape on interventions that have been intentionally implemented with the goal of improving the health and social wellbeing of child brides, who are often at greater vulnerability of experiencing adverse health and social outcomes. While the evidence base is growing on how to improve the sexual and reproductive health (SRH) of adolescents, including on how to prevent adverse outcomes such as unintended pregnancy, HIV, and other morbidities,^11,12^ we know little about how to address the needs of child brides who may be more vulnerable compared to their other adolescent counterparts, or who may face unique challenges in accessing services that meet their specific needs. Similarly, the extent to which existing interventions have been successful in improving the mental health and psychosocial wellbeing of this group is also largely unknown. Despite strong evidence that girls in this group suffer from a range of adverse social outcomes and higher risk of violence, we have little understanding of how to increase social support in this group, or how to ensure that their self-efficacy and sense of autonomy are enhanced, considering their particular circumstances. A systematic examination of the extant literature is thus needed to provide insights on how best to support child brides and improve their health and wellbeing. The need is heightened by the fact that prior to the COVID-19 pandemic, progress would have needed to increase 17-fold to prevent 100 million additional child marriages from taken place by 2030.^13^

To contribute to this evidence base, this paper systematically reviews existing research and documentation of projects and programmes that respond to the health and social needs of married girls in LMICs, where the burden and prevalence are greatest. The overarching research question guiding this review is “What interventions have been implemented to address the health and social needs of child brides and women married as children?”

## Methods

We conducted a systematic scoping review to map the research on interventions addressing child brides, drawing on the methodological framework by Arksey and O’Malley.^14^ Given that research on this topic has been scant, we used this methodology rather than conducting a systematic review which might have limited the studies we included. Instead, we used this methodology to scope the full body of evidence on interventions targeting child brides and identify research gaps. We followed the five stages proposed by this framework:

Stage 1: identifying the research question

Stage 2: identifying relevant studies

Stage 3: study selection

Stage 4: charting the data

Stage 5: collating, summarising and reporting the results

We follow the reporting requirements set out by the Preferred Reporting Items for Systematic reviews and Meta-Analyses extension for Scoping Reviews (PRISMA-ScR) Checklist.

### Data sources

Our review drew on academic and grey literature. We searched a total of seven databases in January 2022 to ensure breadth of coverage: Medline, PubMed, CINAHL, PsycInfo, Global Health, ProQuest and Global Index Medicus. To avoid limiting the search to specific interventions, we scanned a broad literature addressing child brides and thus used search terms that only limited the search to articles addressing this target population, without specifying any search terms specific to interventions. Alternative terms for child brides (such as married adolescents, adolescent brides, youth brides, etc.) were also used to capture a wider body of literature. Below is the search syntax used for Pubmed (the syntax for the remaining databases is included as Supplementary File 1):

married adolescent*[tw] OR adolescent bride*[tw] OR adolescents in union[tw] OR married youth[tw] OR youth bride*[tw] OR youth in union[tw] OR young married[tw] OR young bride*[tw] OR young in union[tw] OR married teen*[tw] OR teen bride*[tw] OR teens in union[tw] OR teenager bride*[tw] OR teenagers in union[tw] OR married child*[tw] OR child bride*[tw] OR children in union[tw]

We also searched reference lists of reviews that were identified through our search strategy, and of select journal articles and selected candidate articles for inclusion.

We scanned the grey literature by searching the websites of 15 key organisations that worked on delaying or mitigating the impact of child marriage. Additionally, we screened the first 1000 hits of a Google search using combinations of the keywords (married, brides, girls, young women, and adolescents).

### Inclusion and exclusion criteria

Studies were eligible if they (i) described an intervention or package of interventions (ii) addressed social and/or health outcomes, (iii) were carried out in low- or middle- income countries as per World Bank classification, (iv) were implemented after 2000 to align with the increasing recognition of child marriage as a critical issue on the development agenda, ^5^ and (v) targeted married adolescents (defined as those aged 10–19 years) or women married as children as part of their target group. If an intervention was targeted to a larger group that included child brides, we included the study only if it presented findings disaggregated by or specific to this age group. For simplicity in this review, we use the term child brides to encompass all females, currently or ever married as children. There were no limitations on the language of studies eligible for inclusion. We excluded posters, commentaries, dissertations, protocols that did not have articles and reviews.

### Article selection

Articles were imported to Rayyan software, and their titles and abstracts were screened for relevance to the aims of the scoping review. Articles that did not meet the inclusion criteria were excluded at this stage. The next stage was a full-text screening, which was followed by data extraction for retained publications. The joint first authors conducted duplicate title and abstract screening as well as full-text screening. Any discrepancies at either stage were addressed during regular meetings between the two authors. Data extraction was conducted by the first three authors in tandem, and each extraction sheet was reviewed by another member of the team to ensure accuracy and consistency. Data were extracted using a standardised data extraction Microsoft Word document, which collected information for each study/project on the following: citation; intervention setting; intervention participants’ characteristics; intentionality of the intervention towards married participants, adolescents, and females; theories and evidence that informed the development of the intervention; intervention duration and frequency; intervention reach and coverage; intervention components; enablers and barriers of implementation; evaluation design and methods; evaluation outcomes; and effect sizes where applicable. The extraction form was pilot-tested by the joint first authors who each independently extracted data from 5 studies, met afterwards to discuss discrepancies, and further refined the form based on a discussion of discrepancies.

### Charting data and analysis

All the articles were read by the joint first authors. Information was collated across the individual data extraction sheets and summarised in a Microsoft Excel spreadsheet. Numerical and thematic analyses were conducted. First, we analysed the types of interventions described, the platform through which they were delivered, and examined theories underpinning these interventions. We classified interventions into one of four delivery platforms: (1) community or home-based interventions; (2) facility-based interventions; (3) school-based interventions; and (4) media-based interventions. We then separately analysed the outcomes investigated in this body of work and grouped them under one of five themes: (1) maternal and reproductive health outcomes; (2) social outcomes; (3) educational outcomes; (4) economic outcomes; and (5) outcomes related to partners and other community members. Findings are reported in narrative synthesis.

## Results

### Description of interventions

**Figure 1.**
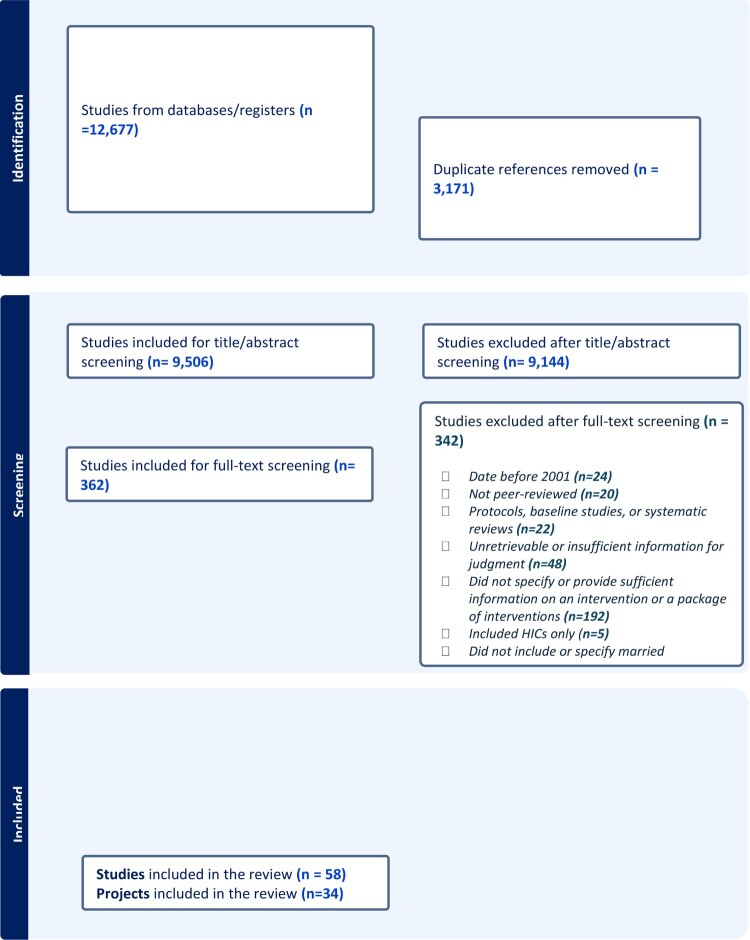
PRISMA diagram

The database search yielded 12,667 titles and abstracts of which only 20 peer-reviewed articles fulfilled eligibility criteria and were included in the review. Another four articles were added as the result of reference search and snowballing. The grey literature search resulted in identifying 34 publications. Articles were organised and grouped under each unique project. Overall, we identified a total of 34 projects that were eligible for inclusion. Figure 1 provides an overview of article selection.

**Figure 2.**
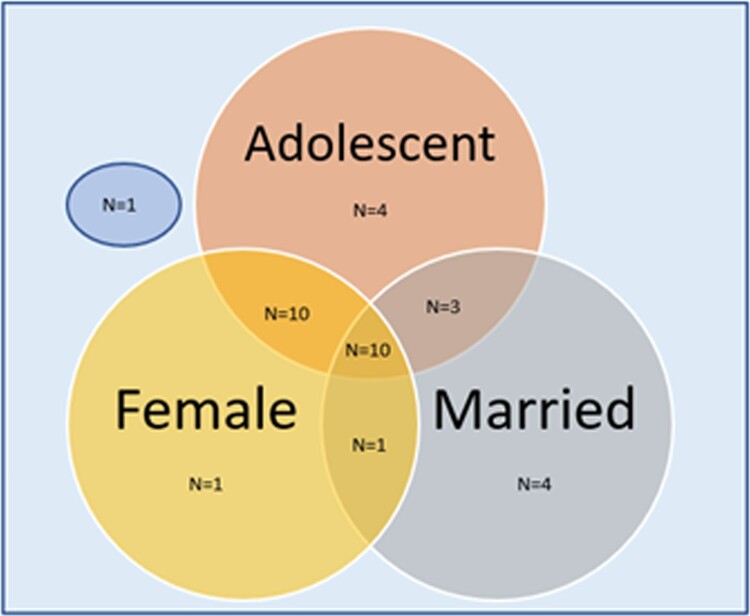
Distribution of included intervention studies by their intentionality to married adolescent girls

Articles on 25 out of the 34 projects were published in 2011 or onwards.^1^ In terms of geographies, South Asia (*n* = 16) and Sub-Saharan Africa (*n* = 15) were most represented in this body of literature, with most projects taking place in India (*n* = 7), Bangladesh (*n* = 7), and Ethiopia (*n* = 5). The Middle East – specifically Iran and Syria – were represented by three projects and Latin America by only one project. Of the 34 projects, 27 described interventions that were specific to adolescents (individuals aged 10–19 years) only, and seven were of interventions that included other age groups (most often young people, individuals aged 10–24 years). Twenty-two focused exclusively on females while 12 included males as well. Eighteen of the included interventions focused on married girls exclusively while 16 included unmarried girls in their target group. Figure 2 shows the distribution of interventions by their intentionality towards married adolescent girls. As demonstrated by the figure, only a small subset (*n* = 10) of interventions exclusively targeted this group. More often, interventions targeted a larger population with married adolescent girls comprising part of the target group. These studies were still included because, in their results, they provided effect sizes that were specific to our interest group – married adolescent girls.

In terms of evaluation design, 19 projects employed mixed methods designs, drawing on both quantitative and qualitative evaluation methods. Fifteen relied on purely quantitative methods, and none of the studies relied exclusively on qualitative data. Of the 34 projects, five were cluster randomised controlled trials, and another five were randomised controlled trials where the unit of analysis was individuals rather than clusters, for a total of 10 projects employing randomisation. Ten projects were quasi-experimental, relying on baseline and endline data as well as a comparison group and another ten used pre/post data without a comparison group. Two relied on endline data alone but used a comparison group as a benchmark, and one collected midline and endline data only and compared outcomes across the two phases of the intervention. Finally, one project drew exclusively on programme data and compared outcomes to national averages for the same population.

### Taxonomy of interventions targeting child brides

Interventions addressing child brides employed various delivery models, integrated different intervention components, and addressed different levels of the social ecology (individual, community, institutional levels). Below we summarise models used to deliver interventions, the types of interventions described, and the theories – if any – that underpinned the design of these interventions. Table 1 presents a summary of the interventions.

**Table 1.** Project Characteristics

Project Title	Country	Setting	Primary participants	Intervention components
Meres Educatrice^52-54^	Burkina Faso’s	Community-based; Facility-based	In-school and out-of-school girls aged 10–19	SRH Education through dialogue and sessions with girls Provision of vitamin A and Iron supplements Escorting girls to health centres for prenatal visits and educational sessions
Meserete Hiwot (Base of Life)^45,55^	Ethiopia	Community-based; Facility-based	Married girls 12–24	• Provided information on communication, self-esteem, reproductive health and gender through girls' groups
Advancing Adolescent Health^56^	Bangladesh	Community-based; School-based; Facility-based	Male and female adolescents ages 10–14 and 15–19 years	Foundational life skills training sessions for adolescents that provided information on SRH matters and negotiation skills to address issues such as age at marriage, delaying births, and other topics Strengthening adolescent-friendly health services and training of healthcare providers in government health facilities
SAFE^35^	Bangladesh	Community-based; Facility-based	Adolescent girls and young women aged 10–29 in the project areas	Access to health and legal services, interactive sessions with men, young women and girls and awareness-raising campaigns in the community Group sessions to discuss sexual harassment, rape, domestic violence and other forms of violence. During sessions, information about locally available services was provided
Relais communautaires^27^	Niger	Community-based	Married adolescent girls aged 13–19	CHWs: r*elais communautaires*, performed a number of tasks related to health promotion, treatment, and linkages with formal health care facilities They are provided with basic pharmaceutical products, family planning commodities, and other first aid equipment that they can then distribute to members of their communities
Acquire I^26^	Bangladesh	Community-based; Facility-based	Married adolescents and young adults	• Integrated capacity building and social change intervention for service providers, social and local leaders, and mothers-in-law to enable the environment for YMC to access SRH care
Acquire II^41^	Nepal	Community-based; Facility-based	Married women <20	• Established a peer education network to disseminate reproductive health information to married couples; supported local health facilities to provide youth-friendly services; and fostered an enabling environment among parents, in-laws, and influential community members to increase married adolescents’ access to, and use of, health services.
Adolescent mothers against All odds^34,42^	Syria	Community-based; Facility-based	First-time mothers and pregnant girls between the ages of 10 and 18 years	The AMAL Initiative has three main components: A Young Mothers’ Club with girls, participatory dialogues with marital family and community members, and reflective dialogues with healthcare providers The Young Mothers’ Club (YMC) is a peer-based discussion group made up of pregnant adolescents and first-time mothers centered around improving sexual and reproductive health knowledge and strengthening life skills
APHIA^20^	Kenya	Community-based; Facility-based; Media-based	Married adolescent girts 14–19 years	An interactive media campaign Training of and following up on four cohorts of community health workers Distribution of RH/FP and HIV information, education, and communication (IEC) materials
Berhane Hewan^24,54,57,58^	Ethiopia	Community-based; School-based	Adolescent girls 10–19 years; married and unmarried	Group formation by adult female mentors Support for girls to remain in school (including an economic incentive), and participation in nonformal education (e.g., basic literacy and numeracy) and livelihood training for out-of-school girls "Community conversations" to engage the community in discussion of key issues, such as early marriage, and in collective problem solving
CMC^33^	India	Community-based	Married women ages 15–30 years and their husbands	• CMC tested two alternate approaches to diagnose and treat reproductive tract infections (RTIs) among rural, young married women ages 15–30 and their partners: reliance on a health aide (Arm A) versus using a female doctor (Arm B)
DISHA^39^	India	Community-based; Facility-based	Married and unmarried male and female youth aged 14–24 years	Improving youth skills and capacity; Youth groups, peer education, income generating opportunities Creating an enabling environment by building community support for meeting youth sexual and reproductive health needs Ensuring youth-friendly sexual and reproductive health service delivery and access
ICRW^44^	Nepal and India	Community-based; Facility-based	Youth aged 14–21 years	Community mobilization with participatory methods: to provide health education on a variety of reproductive health issues to young women, their husbands and mothers-in-law, and the community as a whole More traditional approach to reproductive health: improving the quality of SRH services by training local-level government health functionaries
Functional Analytic Psychotherapy^18^	Iran	Facility-based	Married adolescents 15–19 years	• Primarily Functional analytic psychotherapy with enhanced cognitive therapy (FECT) Sessions that focused on sexual efficacy in addition to cognitive techniques
FRHS^33,44,59^	India	Community-based; Facility-based	Married young women 15–22 years and their husbands	Social mobilization Strengthening health services
GREAT^19,60–62^	Uganda	Community-based; Media-based	Adolescents 10–19	Iterative six phase community mobilization process: Community leaders in each parish identified priority issues in collaboration with their communities, developed a plan to address those issues, carried out the plan and monitored and evaluated their progress Serial Radio Drama Component: The drama included four storylines tailored to VYA, OA, and adults to engage, entertain, inform and spark substantive discussion in communities about gender, violence and SRH including family planning Toolkit Component: including storybooks on puberty for VYA boys and girls, as well as a life-sized board game, radio discussion guides, and activity cards tailored to each life stage. VHT Service Linkages Component: trained village health teams to improve access to and quality of youth-friendly services
Group ANC^17^	Senegal	Facility-based	Adolescents 15–19 (Majority married)	• Four group sessions that promoted clinical follow up and discussions endeavored to enhance knowledge and health literacy that were grounded in a facilitated participatory learning and action approach that promoted peer support
MAG Club^30,31^	Bangladesh	Community-based	Married adolescent girls 15–19	MAG club monthly sessions and curriculum and recreational activities such as dance, music, and drama BRAC health volunteers promote the uptake of long acting reversible and referred MAG to receive LARCs Marriage registrars trained to counsel married couples
IHMP- The Safe Adolescent Transition and Health Initiative (SATHI)^(28,29,63)^	India	Community-based; Facility-based	Married adolescent girls	IHMP works with primary health care workers to identify and assess the health care needs of married girls, to improve married girls’ access to maternal, sexual and reproductive health care services CHW roles: monthly assessment of health, needs, morbidity surveillance, microplanning, need-specific interpersonal communication and counselling, active linkage with health providers, community-based monitoring by village health committees
KEM^33,64^	India	Community-based; Facility-based	Married couples 14 to 25 years	Seven sessions of reproductive health education (RHE) Sexuality counselling sessions for young married couples Clinical referral for those who needed treatment for reproductive morbidities
Marriage: No Child’s Play^38,64,65^	India, Malawi, Mali, Niger, and Pakistan	Community-based; Facility-based	Females 12–19 years	Policy: Advocating for legal frameworks toa dress child marriage Community: creating alternatives for girls instead of marriage Family: working with parents to address gender norms for girls to provide more choice Individual: empowerment programmes that focus on voice and agency
Reach Married Adolescents (RMA)^32^	Niger	Community-based	Young married adolescent girls (ages 13–19), and their husbands	House visits Small group sessions Community dialogue
Sexual and Reproductive Health Counseling^66^	Mexico	Facility-based	Pregnant adolescents aged 16.2 ± 0.66	Training of facilitators by *Ipas Mexico* Instrument based on the perinatal risk cards PREVIGEN IV, V, VI and VIII Preparation of educational material: evidence care towards the end of pregnancy, sexual and reproductive rights, risk factors associated with depression in pregnant adolescents, violence in pregnant adolescents and breastfeeding
Smart Start^67,68^	Ethiopia	Community-based	Adolescent girls 15–19	HEW-initiated community dialogue, support girls and couples with setting goals and help them build a sense of self-efficacy and confidence HEW, alongside a youth Smart Start Navigator connects girls and couples to resources that they need to achieve their goals and raise healthy children HEWs provide contraceptive counselling and services and a Goal Card to help them track their progress against the life goals they map out in their counselling session
TEFSA Bright Future I, II, III^23,40,69,70^	Ethiopia	Community-based	Married adolescents 10–19	Economic Empowerment (EE) – Girls who received economic empowerment information and guidance, based on an adapted VSLA model Sexual & Reproductive Health (SRH) – Girls who learned about issues related to their sexual and reproductive health Combined – Girls who received both EE and SRH programming Comparison – Girls who received a delayed version of the Combined curriculum and served as a comparison group
VSLA^22^	Cote D’Iviore	Community-based	Married females	The group savings model Gender dialogue groups
School support^36^	Zimbabwe	School-based	Married AYA women 17026	• Students in intervention schools received school support, including payment of their school fees, uniforms, exercise books, and other school supplies (e.g., pens, soap, underpants, and sanitary napkins). In addition, a female teacher who was trained by the study to be a “helper” monitored participants’ school attendance and assisted with solving attendance problems
Parenting training^71^	Iran	Facility-based	Married pregnant adolescents, mean age 16+1.12	Three sessions training at weeks 33, 34 and 35 that covers newborn’s characteristics, breastfeeding, warning signs, newborn safety, sleeping among, postnatal care other topics Training activities included: Face-to-face training, vicarious experiences (demonstrating neonatal massage video/modelling on a doll, assessing the mothers’ performances and paying compliments for correct behaviours), discussion and providing CDs and pamphlets
RISE^37^	Zambia	Community-based; School-based	Married and unmarried adolescent girls	Material support such as books pens and pencils to all adolescent girls in the study. The trial also provides monthly cash transfer to some adolescent girls in the study, payment of school fees, and gives money to parents/guardians In addition, other adolescent girls and their communities have community meetings or the parents/guardians, where adolescent girls and boys are invited to attend
Health Boost^21^	Bangladesh	Media-based	Married adolescent girls	• Voice messages with SRH information to enrolled married adolescent girls, twice a week, with re-listening option. Pregnant and non-pregnant participants received different content
COMPASS^72^	Ethiopia	Community-based	Sudanese and South Sudanese 13–19 years old	Weekly adolescent girl life skills sessions in safe spaces, with 45–60 min of facilitated content and 30 min of unstructured time Monthly discussion groups for enrolled girls’ caregivers, which covered topics such as communication skills, supporting adolescent girls and understanding violence and abuse
PRACHAR^15,16,73–76^	India	Community-based; Facility-based	Youth 12–24 years and adult women	Training on sexual and reproductive health and life skills with age-appropriate content for 12–14 and 15–19 age groups, delivered separately to males and females “Newlywed ceremonies” that combined education and entertainment Outreach by female lay health workers “change agents” who conducted home visits and group meetings to counsel and refer women for services at planned intervals timed with life events such as marriage and pregnancy Outreach by male change agents to husbands of young women through regular small-group meetings, which included dialogue and discussion on sexual and reproductive health and gender Home visits and small groups with mothers-in-law, other community outreach activities through street theatre performances, wall paintings, puppet shows, and information, Education and communication (IEC) materials Government and private-sector contraceptive services mapping and strengthening + referrals to these services made by the change agents
KAISHAR^77^	Bangladesh	Community-based; Facility-based	Girls and boys 10–19	• Disseminated information on adolescent reproductive and sexual health to communities, trained health providers to provide services to adolescents, and equipped adolescent information centres
BALIKA^25^	Bangladesh	Community-based;	Girls aged 12 to 18	Education support Life-skills training Livelihoods training

Typically, interventions targeting child brides were bundled, incorporating various intervention components and including different participants such as adolescent girls, their spouses, parents, mothers-in-law and other family members, wider community members and health service providers. Of the 34 projects, 18 were bundled interventions and only 10 delivered a discrete or single component intervention. Eight projects were multi-arm, typically delivering one or more single or bundled intervention to different groups or across different geographies, to determine relative effectiveness. One of the included projects was the PRACHAR project which was unique in that its different phases each entailed a different intervention.^15,16^ In its first phase, the project delivered a bundled intervention that included, among other components, training adolescents on SRH and life skills, outreach visits by lay health workers, small group discussions and meetings with mothers-in-law and other community members, as well as training health providers. In its second phase, the programme attempted to measure the effectiveness of discreet intervention components, and thus utilised a multi-arm design, wherein one arm received home visits, the second arm received the bundled intervention, and the third arm comprised a volunteers-only model. In the third phase, the programme was absorbed by the government and a bundled version of the intervention was delivered.

Interventions included in the review were delivered using various models (as indicated above), which we categorised into the following: (1) community-based interventions, (2) facility-based interventions, (3) school-based interventions and (4) media-based or digital interventions. Because interventions were commonly bundled and addressed multiple actors, they often employed more than one model in combination. Table 2 displays information on intervention delivery platform.

**Table 2.** Intervention models used and illustrative intervention components

Intervention model	Number of studies	Illustrative intervention components
**Exclusively community/home-based**	9	Young mothers’ clubs, married adolescent girl clubs, safe spaces, home visits, community dialogue and sensitisation
**Exclusively health facility-based**	4	Making facilities adolescent-friendly, training of healthcare providers
**Exclusively media-based**	1	Voice messaging for SRH information provision
**Exclusively school-based**	2	Payment of school fees, coverage for costs of uniforms, exercise books, and other school supplies
**Interventions with 2** **+** **delivery models**		** **
**Community-based with facility platform**	16	Youth-focused activities at the community level coupled with strengthening adolescent-friendly health services.
**Community-based with school-based platform**	2	Support for girls to remain in school coupled with community sensitisation
**Community-based with media platform**	2	Serial Radio Drama coupled with training of community-health teams

In total, 27 of the 34 projects had community- or home-based activities. These included education, training, and counselling through peer-led activities, agents of change, home visits by community health workers, and parents’ education. Young mothers’ clubs, Married Adolescent Girl clubs, safe spaces, and other group formation activities were frequently implemented, and participatory and reflective dialogues with girls, boys, their marital and natal families, and wider community members were carried out in several projects. Other community-based activities included distribution of contraceptive methods, vitamin A and Iron supplements and referral to health facilities by community-based workers.

A total of 20 projects included a health facility-based component. Health facility-based activities most commonly included training health providers on adolescent friendly services. The extent to which these services were tailored to the unique needs of married adolescent girls and child brides is not clear, as only some projects mentioned a deliberate emphasis on targeting this group and most did not specify whether training content was tailored specifically to meet the needs of married adolescent girls. Other health facility-based activities included equipping youth-friendly clinics with supplies and Information, Education, and Communication (IEC) material, establishing one-stop service centres, and experimenting with different models of service delivery for adolescents. For example, one project examined the feasibility and effectiveness of group antenatal care,^17^ and another implemented cognitive therapy sessions targeting married adolescent girls to improve their sexual quality of life.^18^

Only three interventions employed a media or digital platform,^19–21^ and only one of those was exclusively digital.^21^ The Health Boost intervention was an innovative health communication intervention which used a voice message system on mobile phones to provide reproductive, maternal and newborn health information to married adolescent girls.^21^ Another bundled intervention included a serial radio drama with themes relevant to gender, violence and SRH in combination with community mobilisation^19^ and one was an interactive media campaign that included a radio soap opera series around the life of a married adolescent girl.^20^

Four projects used schools as their delivery platform. Those typically included differing kinds of support for girls to remain in school, including payment of tuition fees, distribution of books and school supplies, and cash transfers conditional on school attendance. In one intervention, schools were used as a venue for life skills training and after-school homework support.

Most interventions described were primarily SRH-oriented, aiming to improve the sexual and reproductive knowledge, attitudes or practices of child brides through the provision of health services and information. Six projects described interventions with economic components, but those were often folded into a broader health intervention. Examples of projects with an economic component are those described by Falb et al and Edmeades et al which created village savings and loan associations.^22,23^ Other examples are the Balika and Berhane Hewane programmes which implemented livelihood activities among girls.^24,25^ Similarly, very few projects included a legal or advocacy component and those that did also did so in the context of a broader health intervention. One example is the SAFE programme which offered legal services as part of one-stop service centres to survivors of violence.

### Intervention outcomes featured in this body of research

While this body of literature described various intervention modalities and models for delivery, outcomes that were measured were predominantly health-oriented, and specifically related to maternal and reproductive health outcomes. As demonstrated by Table 3 and Supplementary File 2 (which provides detailed information on outcomes measured), a total of 27 articles used maternal or reproductive health outcomes to evaluate intervention effectiveness. Rarely did projects include one primary outcome. Instead, they often evaluated a large combination of outcomes that cut across various categories.

**Table 3.** Outcomes reported in the 34 projects included in the scoping review

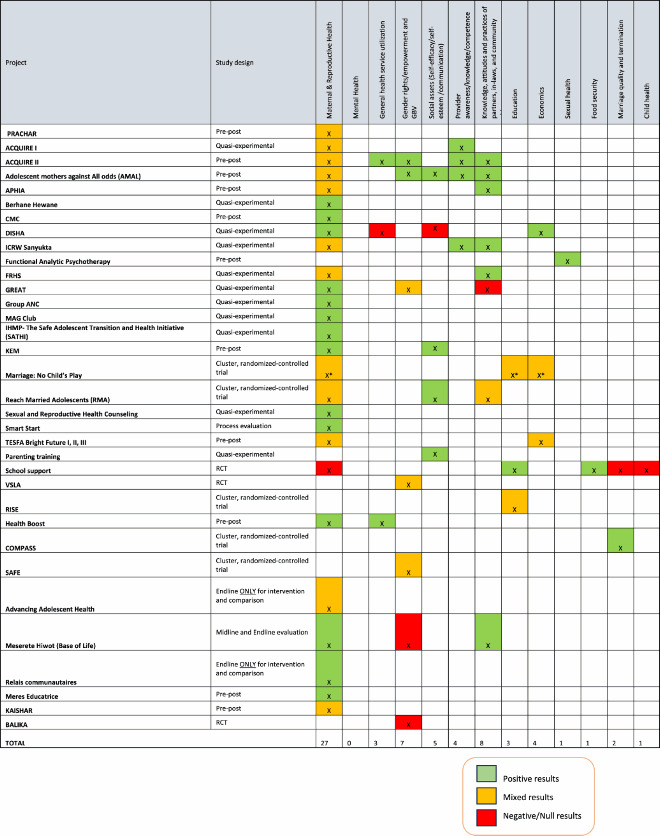

Starkly absent from this literature are interventions that aim to improve mental health outcomes. Only one study addressed the sexual health of married adolescent girls and only two projects looked at outcomes related to marriage quality and marriage termination (divorce or separation). Interventions aiming to improve the health and wellbeing of children of married adolescent girls were also scarce and only one study evaluated impact on a child health-related outcome – immunisation rates among children of child brides. In the following sections, we summarise findings from this body of literature as they pertain to the following categories of outcomes: (1) Maternal and reproductive health outcomes; (2) social outcomes; (3) educational outcomes; (4) economic outcomes; and (5) outcomes related to partners and other community members.

### Maternal and reproductive health outcomes among girls

Projects that investigated maternal and reproductive health outcomes (*n* = 27) often used indicators related to knowledge and attitudes. A smaller subset measured practices and behaviours such as contraceptive uptake, delayed first birth and birth spacing. Several interventions measured utilisation of health services by child brides, specifically antenatal care, delivery and postnatal care. While many interventions were able to change knowledge and attitudes, only eight studies out of the 27 measured and improved practices directly. As shown by the ACQUIRE projects – and specifically ACQUIRE II – changes in knowledge and attitudes measures were more likely to be positive, whereas behavioural measures such as contraceptive use, delay in childbearing, delivery at health centres, median age at first birth were all null.^26^ Studies that showed a positive impact on reproductive health practices had one thing in common: they employed community-based workers who either conducted home visits, convened girl clubs or meetings, or organised community dialogues while simultaneously connecting married adolescent girls to health services. This combination of health promotion and referral to services seems to have been successful in generating gains in reproductive health behaviours. One example is the *Relais Communicatrices* in Niger who performed a number of tasks related to health promotion and treatment, as well as facilitated linkages with formal health care facilities.^27^ Another is the *Shyastha Shebikas* in Bangladesh who were trained to provide comprehensive information about contraception, sell family planning methods and health commodities, and make referrals for LARCs among married adolescent girls who desired to space pregnancies.^28,29^ Yet another is the SATHI programme in India which leveraged CHWs and village health committees to identify and assess health care needs of girls and linked them with health provider.^30,31^

Another promising intervention that showed an impact – though mixed – on reproductive health practices was the PRACHAR intervention in India.^15,16^ While showing mixed effects across study phases and components, the programme showed its greatest positive effect on contraceptive use in its first phase, and positive – albeit milder effects – in its last phase when it was taken to scale. Initially, beginning as a more intensive NGO programme, PRACHAR was eventually absorbed by government systems in an effort to move it to scale. While coverage and scale increased, overburdened government workers and systems may have been responsible for the attenuated impacts of the programme. The programme examined outcomes regarding intervention intensity, timing and scale. For example, the timing of home visits – which were an important component of the programme – was found to affect its success; women who were reached repeatedly across the life cycle at “critical junctures” showed the best outcomes. Intensity of home visits also mattered, and gender synchronisation was key, with programme effects largest when couples were reached together, as compared to when men or women were reached alone. REACH is another programme that showed mixed results across outcomes, but that succeeded in improving contraceptive uptake across all arms.^32^ It used community health workers in Niger (the *relais* mentioned earlier) and compared the effectiveness of (a) home visits to (b) small group sessions to (c) a combined model where both home visits and group sessions were held. The programme documented increases in contraceptive use across all arms, but the highest impact was generated by combined interventions wherein group sessions were followed by home visits. The authors additionally conducted a costing study which showed that, while the combined intervention was more effective, it cost 34% more compared to household visits alone. In contrast, the household visits had the lowest cost of implementation and were highly effective – albeit not as effective as the more costly combined model.

Another promising and cost-effective intervention was CMC, which used community-based approaches to reach young rural married women, specifically deploying village-level female health aides who are trained to undertake speculum exams, examine, and treat women for reproductive tract infections (RTI).^33^ Not only did the intervention engender positive changes in knowledge and practices around RTI treatment, but the authors also made the case for the cost-effectiveness of this approach – illustrating that the use of health aides was both effective and efficient in comparison to using female doctors.

While a number of projects documented health-system interventions to increase married adolescents’ access to, and use of, health services, four of them evaluated intervention outcomes among health service providers themselves. Examples of outcomes assessed include attitudes of service providers (such as their self-reported support for married girls’ reproductive rights, their comfort in delivering family planning services to young girls, among others), their awareness of the special needs of married adolescents and capacity to provide youth-friendly services, and their commitment to assuring the confidentiality of health services. While consistently positive, in one project evaluation, service providers in the intervention voiced contradictory and problematic beliefs wherein they stated that if a girl seeks care without her husband’s presence or permission, they would tell her husband about her visit because they were trained by the programme implementers that family planning should be a “joint responsibility,” involving both men and women.^34^

### Social outcomes

A total of 12 projects assessed social outcomes. Subsumed under social outcomes, we define two categories of outcomes: (1) social assets such as girls’ self-efficacy, self-confidence, negotiation and communication skills; and (2) knowledge and attitudes about gender and girl’s rights, as well as sensitisation around gender-based violence.

Of the five projects that assessed change in social assets, four demonstrated a significant impact. Three of the programmes targeted pregnant young women and first-time mothers, while the other two targeted couples (girls and their husbands). These interventions mostly entailed participatory dialogues with girls or training sessions. The AMAL programme, one of two that were implemented in humanitarian settings, demonstrated a positive increase in self-esteem, confidence in seeking care, and perceived communication ability among married adolescent girls.^34^ Participants noted an increase in “feelings of self-worth, self-respect, and personal happiness”, leadership, communication, and decision-making. However, the programme found that at endline, more girls reported not being able or willing to visit a health facility without their husbands’ permission. The authors hypothesised that this may have been caused by the project’s emphasis on spousal communication, dual engagement, and joint decision-making. However, the authors did not collect data to verify this.

Among interventions that attempted to shift gender attitudes and norms, most projects failed to show an impact. Out of seven interventions, only two generated a positive outcome and the remainder generated either mixed or null findings. The two successful interventions were ACQUIRE II and AMAL; they both assessed self-reported attitudes and norms related to gender, but did not measure behaviours or experiences of gender-based violence. Further, projects that assessed experiences of gender-based violence, such as prevalence of physical and sexual violence or coercion in marriage, were less likely to report significant positive impacts.^22,25,35^

### Education outcomes

Only three projects assessed educational outcomes such as school attendance, school performance, literacy rates, and drop-out rates. A study in Zimbabwe investigating the impact of school support – specifically, payment of school fees, uniforms, and school supplies – found a positive intervention effect among married schoolgirls, with intervention participants accruing one year of education compared to married control participants.^36^ The two other projects showed mixed results with one showing an impact of cash transfers in Zambia on school retention but failing to show an impact of a combined intervention of cash transfers and community dialogues,^37^ a surprising finding that the authors fail to explain, and the second showing impact in two settings (Malawi and India) from among four settings.^2,^^38^

### Economic outcomes

Like educational outcomes, economic outcomes were only assessed in three projects, of which only one study showed a positive intervention impact. The DISHA intervention created income-generating opportunities for married and unmarried young people in Bihar and Jharkhand states and found a positive increase in engagement in livelihood activities.^39^ The multicomponent intervention involved livelihoods training and skills building for young people on how to develop and maintain enterprises, and where possible linked them to micro-savings and credit groups. The two other interventions showed mixed results.

One multi-country intervention increased the proportion of girls working for income in Niger, but failed to produce the same impact in the other three other countries where the intervention was implemented.^38^ The final intervention was the TESFA intervention, which assessed the effectiveness of combined versus single-focus programming, finding a positive impact of the combined programme on two of three economic outcomes (having savings, investing savings, and ability to feed one’s family) and an impact of single programming on only one of the three outcomes.^40^ While the combined intervention generated effects on a larger number of economic outcomes, the more intensive programming in the single-focus programme arm resulted in greater change, particularly on other SRH outcomes that the programme assessed, which led the authors to conclude that while combined programming may lead to improvements across a larger number of outcomes, it may generate lower impact on specific indicators. Programme implementers thus face a trade-off between more intensive impact on a narrower range of outcomes compared to lesser impact on a broader set of outcomes.

### Outcomes specific to husbands, elders and other community members

A subset of projects additionally investigated intervention effects among populations aside from married girls, such as their husbands, in-laws, elders, and community members more broadly.^18,31,32,40–44^ Outcomes generally featured knowledge, attitudes and supportive behaviours among these actors who were assumed to wield great influence on the lives of married girls. Of the eight projects that measured outcomes among these groups, six showed a positive impact.^33,41–45^ Outcomes assessed included attitudes and beliefs of husbands/partners, in-laws, and other community members about reproductive health, family planning, gender norms and decision-making, community tolerance for child and early marriage, and knowledge of modern contraception methods among others. Fewer projects investigated behaviours such as accompaniment to health clinics, or husband’s assistance with domestic chores.

## Discussion

Our review systematically mapped the literature on interventions addressing the health and social needs of child brides. Among the 34 interventions identified, only 10 were intentionally and deliberately tailored to this group. The remaining interventions, while not explicitly designed for child brides, nonetheless presented results for this subgroup, reflecting a growing recognition of their unique needs and vulnerabilities and the imperative for tailored interventions to meet their needs and fulfil their rights. We found that most studies focused on the SRH needs of child brides and leveraged community-based delivery models. This mirrors findings from another review on child marriage prevention and response which underlines the preponderance of SRH interventions compared to interventions that focus on other health outcomes, such as mental and psychosocial health or nutritional status, and interventions that improve social and economic wellbeing.^5^ The sheer number of publications describing SRH interventions has established a critical mass of studies which illustrate the promise of community-based interventions that couple SRH information provision and education with adolescent-friendly health service provision. This finding aligns with other literature that points to the effectiveness of community-based SRH interventions and their linkages to health services among broader age groups. For example, in their 2015 review, Sarkar et al find evidence for the effectiveness of community-based SRH interventions.^46^ Similarly, a qualitative review of modern contraceptive provision concluded that combining provision of information, life skills, support and access to services is key to increasing modern contraceptive method use.^47^ In investigating interventions that reduce rapid repeat pregnancy among adolescents, Norton et al similarly find evidence supporting the combination of clinical and non-clinical interventions.^48^

While the majority of interventions focused on generating demand for SRH services among child brides, a subset of studies in our review described supply-side interventions. Examples include interventions that increase responsiveness of health service providers and health systems to the needs of child brides. Most interventions that were supply-oriented were coupled with community-based demand generation, reflecting the recognition of the importance of combining demand-side with supply-side interventions. While community and health facility delivery models were featured frequently in this body of literature, we note the paucity of media-based or digital interventions – which is surprising given the ubiquity of digital technology in the lives of adolescents. Despite the growing number of studies supporting the effectiveness of digital interventions among adolescents,^49,50^ only three interventions employed a media or digital platform with child brides.

One area where our review failed to provide substantial evidence is marriage dissolution, an important yet understudied topic.^5^ Although our search aimed to identify scholarship targeting child brides and theoretically allowed for the inclusion of articles addressing marriage dissolution, only one study in the evidence base investigated divorce as an outcome. None of the projects described efforts to address the needs of separated, widowed, or divorced girls, who often face stigmatisation and shame. This gap is particularly striking given the growing evidence of the difficulties child brides face in exiting abusive relationships and the lack of interventions to empower them to do so.^51^ These findings highlight the need for future research to better understand and address the intersectional vulnerabilities of child brides, recognising that they are not a homogenous group. Attention to subgroups such as very young married adolescents, child brides with disabilities, and those who are widowed, separated, or divorced is crucial to ensure interventions are appropriately tailored.

Our review should be considered in light of limitations. First, we relied on documents made available online and thus may have failed to capture interventions that were not published on organisations’ websites or in the peer-reviewed literature. Second, we faced difficulties with defining what constitutes an intervention that supports child brides. As articulated in an earlier review by Malhotra and Elnakib, to some degree a broad range of interventions – from employment and cash transfer schemes to family planning and maternal health programmes – that aim to improve the economic, health, social and familial outcomes for married women of all ages, can be said to be mitigating the impact of child marriage since women married as children are frequently participating in such programmes. However, oftentimes these interventions fail to intentionally assess the differential impact on child brides. To achieve conceptual precision, we included interventions that either exclusively targeted child brides or presented stratified analyses that determined specific intervention impact on child brides. Third, we acknowledge the significant heterogeneity in the interventions, contexts and age groups examined. While this scoping review has enabled us to draw some preliminary conclusions, some categories of interventions had too few empirical studies. Our scoping review was intended to provide an initial overview of the landscape and a follow-up systematic review will be necessary as the next step to accurately assess the effectiveness of specific categories of interventions addressing other health, social and economic outcomes once the evidence base of primary studies expand.

Despite these limitations, our findings underscore the need to prioritise the inclusion of child brides in interventions targeting adolescent girls. Restrictions on mobility, increased domestic responsibilities, and heightened vulnerability often exclude child brides from broader initiatives. Tailored interventions are necessary to address these barriers, particularly in settings where child marriage rates remain high.

## Conclusion

The evidence base on interventions for child brides highlights both promising approaches and critical gaps. Interventions addressing the needs of child brides have increased over time, but the preponderance of evidence has focused on SRH interventions, while other areas of health and wellbeing of this group have been overlooked. SRH Interventions leveraging community-based platforms while linking child brides with health services show potential to address unique vulnerabilities. The lack of attention to other health and social outcomes as well as the intersectional vulnerabilities of child brides underscores a pressing need for more targeted research and programming. Future efforts must prioritise child brides as a distinct subgroup, ensuring they are not excluded from broader adolescent initiatives. Addressing these challenges is essential to advancing the health, rights, and well-being of child brides globally.

## Supplementary Material

Supplemtary File 2. Detail of outcomes measured.

Supplementary File 1. Search Syntax

## Footnotes

1 For projects with several publications, we used the date of first publication. One of the projects FTEP, had several reports and publications and spanning four countries with the earliest publication dating back to 2008 and the latest in 2020.

2 The two other countries were Mali and Niger, where there was not a significant impact.

## Abbreviations

Reproductive tract infections (RTI)

Reach Married Adolescents (RMA)

Preferred Reporting Items for Systematic reviews and Meta-Analyses extension for Scoping Reviews (PRISMA-ScR)

## Provenance statement

This article was not commissioned and went through external peer review.
