# Supplementary material for: Mapping the evidence on interventions that mitigate the health, educational, social and economic impacts of child marriage and address the needs of child brides: a systematic scoping review
Source: Sex Reprod Health Matters. 2025 Jan 8;32(1):2449310. doi: 10.1080/26410397.2024.2449310 (PMC12131532; doi:10.1080/26410397.2024.2449310)
Supplement: Supplemtary File 2. Detail of outcomes measured. [file ZRHM_A_2449310_SM4399.docx]

| Project Title | Outcomes: | Study type | Study design | Comparator groups | Intervention effect |
| --- | --- | --- | --- | --- | --- |
| **1.** **PRACHAR** | Current contraceptive use | Quant | Pre-post design  + monitoring data | *Pre and post across several models:*  1. **Phase 1** multicomponent  2. **Phase 2a** single intervention home visits model  3. **Phase 2b** multicomponent model  4. **Phase 2c** single intervention volunteers model  5. **Phase 3** government-NGO model | *Effect on current contraceptive use*  1. Phase 1: **Positive** (aOR=3.84)  2. Phase 2a: **Positive** (aOR of 2.00,P<.01)  3. Phase 2b: **Null**  4. Phase 2c: **Null**  5. Phase 3: **Positive** (aOR of 1.34; P<.01) |
|  |  |  |  | *Selected program components in the difference phases:*  1. Home visits vs no home visits in phase 1  2. Adolescent training + home visits vs either intervention alone in phase 1  3. Small group meetings vs no meetings in phases 1 and 2 | *Effect of selected program components*  1. Home visits: **Positive** aOR=2.30;P<.001  2. Adolescent training + home visits: **Positive** multiplicative effect  3. Small group meetings: **Positive** aOR=3.16;P<.001 |
| **2. ACQUIRE I** | - Contraceptive prevalence  - Attitudes about timing of first birth  - Health center visits  - Knowledge about  required ANC and use  - Tetanus toxoid coverage  - Delivery at health centers  - Knowledge about the danger signs of pregnancy, delivery, and postpartum period  - Knowledge about STIs, symptoms of STIs, and how to avoid STI  - Attitudes about family planning decision-making | Mixed | Quasi-experimental + interviews with providers | Baseline vs endline in intervention vs control group | - Contraceptive prevalence: **Positive**  - Attitudes about timing of first birth: : **Positive**  - Health center visits: **Positive**  - Knowledge about  required ANC: : **Positive but not accompanied with increase in ANC use**  - Tetanus toxoid coverage: **Null**  - Delivery at health centers: **Negative**  - Knowledge about the danger signs of pregnancy, delivery, and postpartum period: **Positive**  - Knowledge about STIs, symptoms of STIs, and how to avoid STI: **Positive**  - Attitudes about family planning decision-making: **Positive**  - Attitudes of service providers: **Positive** |
|  | - Attitudes of service providers |  |  |  |  |
| **3. ACQUIRE II** | - Use of Health Services  - Knowledge of modern contraceptives  - Attitudes about timing of first birth  - Attitudes about family planning decision-making  - Use of contraception before first pregnancy  - Knowledge of danger signs during pregnancy, delivery, and postpartum  - Knowledge and use of antenatal care  - Birth planning and use of delivery and post-natal care  - Delay in childbearing  - Knowledge of HIV/AIDS and STIs  - Perceptions of the ideal age of gauna and motherhood  - Gender Attitudes  - Median age at marriage  - Median age at *gauna*  - Mean duration of the gap between *gauna* and the birth | Mixed | Pre-post design  + interviews with peer educators + FGDs with mothers in law and husbands + FGD with health facilitators | Baseline vs endline | - Delay in childbearing: **Null**  - Use of contraception before first pregnancy: **Null**  - Use of Health Services: **Positive**  - Knowledge of modern contraceptives: **Positive**  - Attitudes about timing of first birth: **Positive**  - Attitudes about family planning decision-making: **Positive**  - Knowledge of danger signs during pregnancy, delivery, and postpartum: **Null**  - Knowledge and use of antenatal care: **Positive**  - Birth planning and use of delivery and postnatal care: **Positive**  - Knowledge of HIV/AIDS and STIs: **Positive**  - Perceptions of the ideal age of gauna and motherhood: : **Positive**  - Gender Attitudes: **Positive**  -Median age at marriage: **Positive**  - Median age at *gauna* : **Positive**  - Median age at first birth: **Null**  - Mean duration of the gap between *gauna* and the birth: **Positive** |
|  | - Providers’ awareness of the special needs of married adolescents  - Provider responsiveness |  |  |  | - Providers’ awareness of the special needs of married adolescents: **Positive**  - Provider responsiveness: **Positive** |
|  | - Mothers in law reproductive health attitudes and behavior |  |  |  | - Mothers in law reproductive health attitudes and behavior: **Positive** |
| **4. Adolescent mothers against All odds (AMAL)** | - Self-esteem  - Confidence in seeking care  - Perceived communication ability  - Perceived relationships with family members  - Leadership capacity  - Beliefs about social norms around education, and use of family planning without husband’s permission  - Individual and community tolerance for child and early marriage. | Mixed | Pre-post design + focus group discussions. | Baseline vs endline | - Self-esteem: **Positive**  - Confidence in seeking care: **Positive**  - Perceived communication ability: **Positive**  - Perceived relationships with family members: **Positive**  - Leadership capacity: **Positive**  - Beliefs about social norms around use of family planning without husband’s permission: **Null**  - Individual and community tolerance for child and early marriage: **Positive** |
| **5. APHIA** | - Use of family planning methods  - Partner support for use of RH services  - Partner support for ANC and delivery services  - Attendance of fur antenatal care visits  - Births at facilities  - Skilled attendant at birth  - Use of postnatal care services within the first 48 hours of delivery  - Acceptance of a FP method postpartum  - Married adolescent girls and partners that worry they might be HIV-positive  - Obtaining an HIV test | Quant | Pre-post design | Baseline vs endline | - Use of family planning methods: **Positive**  - Partner support for use of RH services: **Positive**  - Partner support for ANC and delivery services: **Positive**  - Attendance of the four : **Positive** recommended antenatal care visits  - Births at facilities: **Positive**  - Skilled attendant at birth: **Null**  - Use of postnatal care services within the first 48 hours of delivery: **Positive**  - Subsequent postnatal care visits: **Null**  - Proportion of those that accepted an FP method postpartum: **Null**  - The proportion of married adolescent girls and partners that worry they might be HIV-positive: **Positive**  - Obtaining an HIV test: **Positive** |
| **6. Berhane Hewane** | - Contraceptive use. | Quant | Quasi-experimental | Baseline vs endline in intervention vs control group | - Contraceptive use: **Positive** |
| **7. CMC** | - Prevalence of select reproductive tract infections (RTIs)  - Treatment for partners of women with RTI symptoms | Mixed | Pre-post without a control^[[1]](#footnote-1)^ with qual methods | Baseline vs endline and Arm 1 (health aides) vs Arm 2 (female doctors) | - Prevalence of six RTIs for which lab tests were conducted at endline vs baseline: **Positive**  - Treatment for symptomatic women in arm 1 vs arm 2: **Positive**  **-** Knowledge of 3+ RTI symptoms at endline compared to baseline: **Positive** |
| **8. DISHA** | Youth-level outcomes:   1. Knowledge & Attitudes 2. Youth Empowerment 3. Livelihoods Engagement (only qual) 4. Contraceptive use   Community-Level Outcomes   1. Uptake of services from Youth Depot holders | Mixed | Quasi-experimental with qual methods | Baseline vs endline in intervention vs control group | Youth-level outcomes:   1. Knowledge & Attitudes: Positive 2. Youth Empowerment: Null 3. Livelihoods Engagement (only qual): Positive 4. Contraceptive use: Positive   Community-Level Outcomes   1. Uptake of services from Youth Depot holders: Quant data 🡪 Null; Qual data 🡪 some awareness and use of DISHA-related providers had begun |
| **9. ICRW Sanyukta** | *Women-related outcomes:*   1. Knowledge of maternal care 2. Utilization of MH services   *Partner and elder-related outcomes:*   1. Changed Attitudes of partners and elders 2. Increased Support of Partners and Elders   Health provider outcomes:   1. Capacity to provide youth-friendly services | Mixed | Quasi-experimental with qual methods | Baseline vs endline in intervention vs comparator group (comparator group implemented a more traditional approach to RH) | *Women-related outcomes:*  1. Knowledge of maternal care   - Knowledge of ANC: **Null** - Complications during delivery: **Negative** - Knowledge of PNC: **Positive**   2. Utilization of MNH services   - ANC use: **Positive** - Deliveries in a medical facility, and deliveries attended by a health professional: **Positive** - Use of PNC**: Negative**   *Partner and elder-related outcomes:*   1. Changed Attitudes of partners and elders   1. Increased Support of Partners and Elders: **Positive**  *Health provider outcomes:*  1. Capacity to provide youth-friendly services: **Positive** |
| **10. Functional Analytic Psychotherapy** | Sexual quality of life and its dimensions | Quant | Pre-post design | Baseline vs endline | Mean score of sexual quality of life : **Positive** |
| **11. FRHS** | *Women-related outcomes:*  Women’s knowledge and use of services for maternal health (antenatal, delivery and postnatal), contraceptive use, abortion, infertility and treatment of reproductive tract infection (RTI) symptoms  *Partner and elder-related outcomes:*  Husbands’ knowledge of, and participation in, their wives’ health seeking and the attitudes of mothers-in-law | Mixed | Quasi-experimental with qualitative methods | Baseline vs endline in four arms –  Arm 1: social mobilization  Arm 2: Strengthening government services  Arm 3: Both  Arm 4: None | *Arms 1 vs 2:*  1. Knowledge of maternal care: **Positive**  2. Postnatal check-ups: **Positive**  3. Contraceptive use: **Positive**  4. Treatment of gynecological disorders: **Positive**  5. Partner treatment for symptoms of RTIs or STIs: **Positive**  Overall, the social mobilization arm performed better than the government services arm  For other arms, mixed evidence (varies by outcome) but arm 1 still did better on a range of outcomes.  *Partner and elder-related outcomes:*  Husbands’ knowledge of, and participation in, their wives’ health seeking and the attitudes of mothers-in-law: **Positive** |
| **12. GREAT** | - Gender-equitable values, attitudes, and behaviors among adolescents age 10-19 and adults;  - SRH knowledge, attitudes, and access to services among adolescents 10-19;  - Tolerance of GBV among adolescents and significant others. | Mixed | Quasi-experimental with qualitative methods | Baseline vs endline and control group | - Gender-equitable values, attitudes, and behaviors among adolescents age 10-19 and adults: **Positive**  - SRH knowledge, attitudes, and access to services among adolescents 10-19: **Positive**  - Tolerance of GBV among adolescents and significant others: **Null**  - GBV experiences: **Mixed** |
| **13. Group ANC** | Acceptability and preference for Group ANC vs individual ANC | Mixed | Quasi-experimental with qualitative methods | Group ANC vs Individual ANC | Acceptability and preference for Group ANC vs individual ANC: **Positive**  Qualitative findings indicated key facets of consideration relevant to G-ANC for adolescents include social connectedness, the influence of social norms and the opportunity for engagement in healthcare. |
| **14. MAG Club** | -Knowledge of modern FP methods  - Current use of modern method  - Support for use of FP methods  - Husband support for use of FP methods  - Responsibility of FP is shared between husbands and wives  - Knowledge of consequences of early pregnancy | Mixed | Quasi-experimental with qualitative methods | Baseline vs endline in intervention vs control group | -Knowledge of modern FP methods: **Positive**  - Current use of modern method: **Positive**  - Support for use of FP methods: **Positive**  - Husband support for use of FP methods: **Positive**  - Responsibility of FP is shared between husbands and wives: **Positive** |
| **15. IHMP- The Safe Adolescent Transition and Health Initiative (SATHI)** | - Median age at first birth  - Contraceptive use  - Treatment use for reproductive tract infection  - Testing for HIV  - Antenatal care, delivery and postnatal services | Quantitative | Quasi-experimental | Baseline vs endline in intervention vs control group | - Median age at first birth: **Positive**  - Contraceptive use: **Positive**  **-** Treatment use for reproductive tract infection or sexually transmitted infection: **Positive**  **-**Testing for HIV**: Positive**  *Antenatal care, delivery and postnatal services*  - Early antenatal registration: **Positive**  **-** Minimum standard antenatal care: **Positive**  - Treatment for antenatal complications: : **Positive**  - Treatment for postnatal and neonatal complications: **Positive**  - Increase in Institutional delivery: **Positive** |
| **16. KEM** | - Increase in referrals for clinical  - Changes in reproductive health knowledge specifically knowledge of pregnancy, contraception and risky sexual behavior.  - Couple communication | Mixed | Pre-post with qual methods | Baseline and endline | - Increase in referrals for clinical: **Positive**  - Couple communication: **Positive**  - RH knowledge of menstruation, delivery, contraception and abortion: **Positive**  - Knowledge about ANC: **Positive**  - Knowledge of danger signs: **Positive**  -Knowledge about STIs, HIV, and risky sexual behavior: **positive** |
| **17. Marriage: No Child’s Play** | Schooling, work, and indicators measuring SRH knowledge and attitudes. | Mixed | Cluster randomized design (India, Malawi) & quasi-experimental matched design (Mali, Niger) + Qual methods | Baseline vs endline in intervention vs control group in each of four study settings | - Proportion of girls currently working for income: **Positive** in Niger  - Proportion of girls who had ever attended school: **Positive** in Malawi and India  - Mean number of years of education completed, and decreasing illiteracy rates among 12–19-year-old girls: **Positive** in Malawi  - Girls’ engagement in groups, clubs, or associations.: **Positive** in India  *Knowledge and attitudes*  - Proportion of girls with knowledge about HIV: **Positive** in India  - Proportion of girls with knowledge about modern contraceptives : **Positive** in Niger |
| **18. Reach Married Adolescents (RMA)** | - **Females**: SRH knowledge, attitudes, norms, Self-efficacy, intention to use and actual use  - **Males**: SRH knowledge, attitudes, norms, self-efficacy, among males | Mixed | Cluster, randomized-controlled trial | Arm 1: House visits  Arm 2: Small group sessions  Arm 3: Both  Arm 4: Control | *Females*  - Knowledge of modern contraception: **Positive in all arms**  **-** Attitudes supportive of contraception: only **Positive** in arm 1  **-** Norms**: Positive** only arms 1 and 3  - Self-efficacy: **Positive** **in all arms**  - Intention to use: **Positive** **in all arms**  **-**Actual use: **Positive** in all arms but strongest in Arm 3  *Males*  - Knowledge of modern contraception: **Positive** in all arms  **-** Attitudes supportive of contraception among males: **Positive** in Arm 1 and Arm 2; **Null** in Arm 3  - Norms: **Positive** in Arm 1 and Arm 2; **Null** in Arm 3  - Self-efficacy: **Positive** in all arms |
| **19. Sexual and Reproductive Health Counseling** | Risks in pregnant adolescents during delivery, postpartum and neonate. | Quant | Quasi-experimental | Intervention vs control group | Obstetric and neonatal risks: **Positive** |
| **20. Smart Start** | Use modern methods, LARCS, and method mix | Quant | Process evaluation | Intervention vs national average for this population | Use modern methods, LARCS, and method mix: P**ositive** |
| **21. TESFA Bright Future I, II, III** | - Income, savings and use of loans  - Girls’ savings behavior  - Control over economic decisions  - SRH knowledge  - Contraceptive Use  - Knowledge of STIs  - Changing Views of Contraception- SRH Decision making and communication | Mixed | Pre-post with qual methods | Economic Empowerment (EE)  Sexual & Reproductive Health (SRH)  Combined  Comparison group | Participation in either intervention arm significantly improved four of the five SRH outcomes, with the largest gains in the single-focus arm.  In contrast, those girls in the combined arm experienced improvements in two of the economic outcomes compared with one in the single-focus arm. |
| **22. Parenting training** | Self-efficacy  Bonding scores  Social support score | Quant | Quasi-experimental | Baseline vs endline in intervention vs control group | - Self-efficacy**: Positive**  Bonding scores**: Positive**  Social support score **: Positive** |
| **23. School support** | - Schooling  -Happiness in marriage  - Polygamous marriages  - Family planning  - Child immunization  - HIV testing  - Food security | Mixed | RCT | Intervention vs Control group | - Schooling: **Positive**  -Happiness in marriage: **Negative**  - Polygamous marriages: **Null**  - Family planning: **Negative**  - Child immunization: N**egative**  - HIV testing: **Negative**  - Food security: **Positive** |
| **24. VSLA** | - Past-year physical and/or sexual IPV  - Economic abuse | Quant | RCT | Arm 1: VSLA  Arm 2: VSLA + GDG group | **Arm 2 vs Arm 1**  - Past-year physical and/or sexual IPV: **Null**  - Economic abuse: **Positive** |
| **25. RISE** | School dropout | Quant | Cluster Randomized Controlled Trial (RCT) | Arm 1:  Cash transfer arm  Arm 2: Combined cash and community dialogue arm  Arm 3: Control | Arm 1 vs Arm 3: **Positive**  Arm 1 vs Arm 2: **Null** |
| **26. Health Boost** | - Knowledge of danger signs during pregnancy , child delivery , and for newborns  - Utilization of services  - Utilization of ANC  - Use of PNC | Quant | Pre-post design | Baseline and Endline | - Knowledge of danger signs during pregnancy, child delivery , and for newborns: **Positive**  - Child delivery: **Positive**  - Utilization of services: **Positive**  - Utilization of ANC: **Positive**  - Use of PNC: **Positive** |
| **27. COMPASS** | - Marriage termination | Quant | Cluster Randomized Controlled Trial (RCT) | Baseline vs endline in intervention vs control group | - Marriage termination: **Positive** |
| **28. SAFE** | - Prevalence and severity of physical and sexual violence  - Prevalence of economic violence | Mixed | Cluster Randomized Controlled Trial (RCT) + qual methods | Arm 1: Group sessions among men and women  Arm 2: Group sessions with women only  Arm 3: No group session (Arm C) | - Prevalence and severity of physical and sexual violence: **Positive** in Arms 1 and 2  - Economic violence: **Positive** in arm 1 but **Negative** in Arm 2 |
| **29. Advancing Adolescent Health** | - Preference to delay first birth  - Preference to space between the first and second births  - Knowledge of at least three modern family planning (FP) methods  - Knowledge of at least one source of FP methods  - Contraceptive use | Mixed | Endline ONLY for intervention and comparison group | Intervention vs comparison group | - Preference to delay first birth : **Positive**  - Preference to space between the first and second births: **Null**  - Knowledge of at least three modern family planning (FP) methods: **Null**  - Knowledge of at least one source of FP methods: **Null**  - Contraceptive use: **Null** |
| **30. Meserete Hiwot (Base of Life)** | - Husbands' assistance with domestic work,  - Accompaniment to the clinic,  - Family planning use  - Voluntary counseling and testing (VCT)  -Domestic violence | Quant | Midline and Endline evaluation (NO BASELINE) | Exposure vs no exposure at endline | - Husbands' assistance with domestic work: **Positive**  - Accompaniment to the clinic: **Positive**  - Family planning use: **Positive**  - Voluntary counseling and testing (VCT): **Positive**  -Domestic violence: **Null** |
| **31. Relais communautaires** | Current use of modern contraceptive methods | Quant | Endline ONLY for intervention and comparison group | Exposure vs no exposure at endline | - Current use of modern contraceptive methods: **Positive** |
| **32. Meres Educatrice** | - Adolescents’ knowledge of obstetric fistula  - Adolescents’ knowledge of means to avoid pregnancy,  - Adolescents’ use of sexual and reproductive health services | Quant | Pre-post design | Baseline and Endline | - Adolescents’ knowledge of obstetric fistula : **Positive**  - Adolescents’ knowledge of means to avoid pregnancy: **Positive**  - Adolescents’ use of sexual and reproductive health services: **Positive** |
| **33. KAISHAR** | Awareness about reproductive health   Knowledge of reproductive health problems  Knowledge of sources of RH care services  Actual age at first pregnancy  Actual age at first birth  Reported use of any FP method  Reported use of modern methods  Receiving TT vaccine  Knowledge of HIV/AIDS and mode of transmission  Knowledge of STIs  Knowledge of STI mode of transmission | Quant | Pre-post  Stratified, two-stage cluster sampling | Baseline and endline (no control group) | Awareness about reproductive health: **Positive**   Knowledge of reproductive health problems: **Null**  Knowledge of sources of RH care services: **Positive**  Actual age at first pregnancy : **Positive**  Actual age at first birth: **Positive**  Reported use of any FP method: **Positive**  Reported use of modern methods: **Positive**  Receiving TT vaccine: **Positive**  Knowledge of HIV/AIDS and mode of transmission: **Null**  Knowledge of STIs: **Null**  Knowledge of STI mode of transmission: **Positive** |
| **34. BALIKA** | Marriage choice and consent  School attendance | Mixed | RCT | ARM1: educational tutoring  ARM2: gender rights awareness, ARM3: livelihoods training (computers, mobile phone entrepreneurship, servicing, photography, basic first aid. )  A control area. | Marriage choice and consent (several indicators): **Mostly null** with the exception that girls in the gender-rights (arm 2) awareness intervention were significantly more likely to say no dowry was demanded |

1. There was initially a control group but it was dropped [↑](#footnote-ref-1)
