# Supplementary material for: Mapping the evidence on interventions that mitigate the health, educational, social and economic impacts of child marriage and address the needs of child brides: a systematic scoping review
Source: Sex Reprod Health Matters. 2025 Jan 8;32(1):2449310. doi: 10.1080/26410397.2024.2449310 (PMC12131532; doi:10.1080/26410397.2024.2449310)
Supplement: Supplementary File 1. Search Syntax [file ZRHM_A_2449310_SM4398.docx]

Search syntax

Medline
((child* or young or adolescen* or youth or teen*) adj2 (married or bride? or "in union" or "in-union"))

Pubmed

married adolescent*[tw] OR adolescent bride*[tw] OR adolescents in union[tw] OR married youth[tw] OR youth bride*[tw] OR youth in union[tw] OR young married[tw] OR young bride*[tw] OR young in union[tw] OR married teen*[tw] OR teen bride*[tw] OR teens in union[tw] OR teenager bride*[tw] OR teenagers in union[tw] OR married child*[tw] OR child bride*[tw] OR children in union[tw]

CINAHL

TI ( ((child* or young or adolescen* or youth or teen*) N2 (married or bride$ or "in union" or "in-union") ) OR AB ( ((child* or young or adolescen* or youth or teen*) N2 (married or bride$ or "in union" or "in-union") ) OR MW ( ((child* or young or adolescen* or youth or teen*) N2 (married or bride$ or "in union" or "in-union") )

Global Health

title:((married adolescent*) OR (adolescent bride*) OR (young married) OR (young bride*) OR (teen bride*) OR (teens in union) OR (teenager bride*) OR (teenagers in union) OR (child bride*) ) OR ab:((married adolescent*) OR (adolescent bride*) OR (young married) OR (young bride*) OR (teen bride*) OR (teens in union) OR (teenager bride*) OR (teenagers in union) OR (child bride*) ) OR de:((married adolescent*) OR (adolescent bride*) OR (young married) OR (young bride*) OR (teen bride*) OR (teens in union) OR (teenager bride*) OR (teenagers in union) OR (child bride*) )

((child* or young or adolescen* or youth or teen*) AND (married or bride? or "in union" or "in-union")

GMI

(married adolescent*) OR (adolescent bride*) OR (young married) OR (young bride*) OR (teen bride*) OR (teens in union) OR (teenager bride*) OR (teenagers in union) OR (child bride*)

PsycInfo
((child* or young or adolescen* or youth or teen*) N3 (married or bride? or "in union" or "in-union"))

ProQuest Central

(pub(program* OR intervention* OR project* OR initiative* OR service* OR approach* OR pilot* OR evaluat* OR feasibility OR experiment* OR randomi* OR quasiexperiment* OR control OR trial* OR comparison OR outcome* OR output* OR impact OR effectiveness OR efficacy) OR ab(program* OR intervention* OR project* OR initiative* OR service* OR approach* OR pilot* OR evaluat* OR feasibility OR experiment* OR randomi* OR quasiexperiment* OR control OR trial* OR comparison OR outcome* OR output* OR impact OR effectiveness OR efficacy) OR mainsubject(program* OR intervention* OR project* OR initiative* OR service* OR approach* OR pilot* OR evaluat* OR feasibility OR experiment* OR randomi* OR quasiexperiment* OR control OR trial* OR comparison OR outcome* OR output* OR impact OR effectiveness OR efficacy)) AND ((pub((married adolescent*) OR (adolescent bride*) OR (young married) OR (young bride*) OR (teen bride*) OR (teenager bride*) OR (child bride*)) OR ab((married adolescent*) OR (adolescent bride*) OR (young married) OR (young bride*) OR (teen bride*) OR (teenager bride*) OR (child bride*)) OR mainsubject((married adolescent*) OR (adolescent bride*) OR (young married) OR (young bride*) OR (teen bride*) OR (teenager bride*) OR (child bride*))) AND stype.exact("Scholarly Journals"))
